# *Potentilla parvifolia* strongly influenced soil microbial community and environmental effect along an altitudinal gradient in central Qilian Mountains in western China

**DOI:** 10.1002/ece3.10685

**Published:** 2023-11-14

**Authors:** Miaomiao Cheng, Jinge Song, Weikun Li, Yiming Zhao, Gaosen Zhang, Yong Chen, Haining Gao

**Affiliations:** ^1^ College of Life Sciences and Engineering, Hexi University, Key Laboratory of the Hexi Corridor Resources Utilization of Gansu Zhangye China; ^2^ School of Life Sciences Lanzhou University Lanzhou China; ^3^ School of Stomatology Lanzhou University Lanzhou China; ^4^ Key Laboratory of Extreme Environmental Microbial Resources and Engineering Lanzhou China

**Keywords:** environmental effect, *Potentilla parvifolia*, Qilian Mountains, soil microbial communities

## Abstract

The Qilian Mountains (QLMs) form an important ecological security barrier in western China and a priority area for biodiversity conservation. *Potentilla parvifolia* is a widespread species in the mid‐high altitudes of the QLMs and has continuously migrated to higher altitudes in recent years. Understanding the effects of *P. parvifolia* on microbial community characteristics is important for exploring future changes in soil biogeochemical processes in the QLMs. This study found that *P. parvifolia* has profound effects on the community structure and ecological functions of soil microorganisms. The stability and complexity of the root zone microbial co‐occurrence network were significantly higher than those of bare soils. There was a distinct altitudinal gradient in the effect of *P. parvifolia* on soil microbial community characteristics. At an elevation of 3204 m, *P. parvifolia* promoted the accumulation of carbon, nitrogen, and phosphorus and increased sucrase activity and soil C/N while significantly improving the community richness index of fungi (*p* < .05) compared with that of bacteria and the relative abundance of *Ascomycota*. The alpha diversity of fungi in the root zone soil of *P. parvifolia* was also significantly increased at 3550 m altitude. Furthermore, the community similarity distance matrix of fungi showed an evident separation at 3204 m. However, at an altitude of 3750 m, *P. parvifolia* mainly affected the bacterial community. *Potentilla parvifolia* increased the bacterial community richness. This is in agreement with the findings based on the functional prediction that *P. parvifolia* favors the growth and enrichment of denitrifying communities at 3550 and 3750 m. The results provide a scientific basis for predicting the evolutionary trends of the effects of *P. parvifolia* on soil microbial communities and functions and have important implications for ecological governance in the QLMs.

## Graphical abstract

This study found that the effect of *P. parvifolia* on the soil microbial community has an obvious altitudinal gradient effect. At 3204 m, it mainly affected the diversity and richness of the fungi. However, at 3550 and 3750 m, the effect on bacteria was more pronounced.

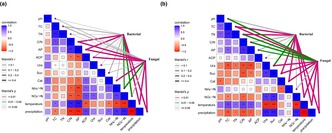

## 1 INTRODUCTION

The Qilian Mountains (QLMs) are located in the northeast of the Qinghai–Tibet Plateau and span the Gansu and Qinghai provinces. This region is the birthplace and water supply area of the inland river basin in the Hexi Corridor and is an important ecological barrier in China (Barberán & Bates, 2012; Dawen et al., 2019; Shouguo et al., 2022). In recent years, with the enhancement of understanding of the unique geographical location of the QLMs, research on soil microorganisms in this region has gradually expanded, and progress has been made in altitudinal gradients (Fan et al., 2022). The altitudinal gradient covers the comprehensive impact of various environmental factors, such as temperature, precipitation, and light intensity, and plays an important role in determining the vertical distribution pattern of species diversity (Lomolino, 2001; Zhang, Wang, et al., 2017).

Shrubs play an important role in water conservation and river runoff regulation. They are also important carbon sinks in forest and mountain ecosystems and play an important role in regional ecological environment protection (Seta et al., 2019; Yayneshet & Treydte, 2015). Having well‐developed root systems and high stress resistance, *Potentilla parvifolia*, a shrub tree species, maintains the soil and conserves water and is widely distributed in the Gansu and Qinghai regions of China being the dominant shrub plant in the QLMs (Barkley & Arthur Cronquist, 1996; Gregory et al., 2010). *Potentilla parvifolia* is mainly distributed in the mid‐high altitudes of the QLMs (Yanyan et al., 2022). Warming experiments have shown that the growth and abundance of *P. parvifolia* in alpine meadows has increased under climate change in cold regions (Elmendorf et al., 2012; Klein et al., 2007) and that they tend to migrate to higher latitudes.

Plants change the nutritional status of soil by secreting litter and root exudates, thereby changing the structure and function of soil microbial communities. Plant litter is an important habitat for microorganisms (Fenglian et al., 2017). During soil microbial succession, the microbial community structure is formed, which plants play a role in, and is further stabilized by interactions with the plant (Johanna & Beat, 2018). Wardle (2006) found that the input of different plants to underground resources can affect the composition of root zone microbial communities, and subsequently, the transformation of functional groups. Few studies have investigated the effects of *P. parvifolia* on soil physical and chemical properties and microbial ecological function (Bardgett et al., 2005), and the redox reactions of carbon and nitrogen cycles are an important topic of study. In recent years, a prominent feature of global climate change caused by human activities has been the increase in nitrogen deposition (Kwaku et al., 2021). The soil nitrogen cycle is an important component of elemental cycling in terrestrial ecosystems and is mainly driven by soil microorganisms (Wang & Zou, 2020), but this process in the plant root zone relies on interactions between plant root exudates and microorganisms. Denitrification is one of the major pathways that contribute to the loss of soil N to the atmosphere and thus has a high impact on environmental change (David et al., 2013; Xiaoxuan et al., 2022; Yin et al., 2014). Predicting the effects of *P. parvifolia* on soil denitrification under climate change is key to assessing the ecological functions of soils in the QLMs.

Soil microbes maintain aboveground interactions in terrestrial ecosystems and are important drivers of plant diversity and productivity. Their diversity and abundance play key roles in regulating ecosystem functions, such as the soil microenvironment and nutrient cycling (Bardgett & van der Putten, 2014; Mishra et al., 2012). Therefore, improving our understanding of microbial communities is important, particularly regional microbial distribution patterns (Franklin & Mills, 2003). Several important abiotic and biotic factors drive changes in microbial composition and diversity, including pH, climate, soil nutrients, plant diversity, and soil enzyme activity (de Vries et al., 2012; Nemergut et al., 2010; Scheibe et al., 2015). These factors can be summarized into two scale‐dependent categories: (1) regional climate conditions, particularly precipitation and temperature (Singh et al., 2014), and (2) local soil conditions, such as soil pH and nutrient content (Birkhofer et al., 2008; Shen et al., 2013). Although the distribution patterns of microbial communities have been previously researched at the global scale (Maestre et al., 2015; Mohammad et al., 2018), there is still a lack of understanding regarding the distribution of microbial communities at the local scale and their drivers (Feng et al., 2019).

To reveal the complex interactions among microbial communities, co‐occurrence networks have been widely used (Bartram et al., 2014; Hartmann et al., 2015), which can reflect ecological relationships and processes that species diversity cannot reflect (Barberán & Bates, 2012). Wagg et al. (2019) confirmed that soil microbial network complexity plays an active role in maintaining ecosystem function through an indoor microbial diversity control trial. The analytical method of inferring co‐occurrence networks between microbial communities from data obtained using second‐generation sequencing technology is widely used in microbial ecology (David & Stefanie, 2014; Siles et al., 2021). In addition, microbial co‐occurrence networks allow the prediction of keystone species and potential species interactions (Fuhrman, 2009; Ye et al., 2012).

In this study, we used a new‐generation sequencing method based on 16S/ITS rRNA to reveal soil microbial diversity, community composition, and ecological networks at an altitude of 3204–3750 m in the Binggou watershed of the QLMs. The aims of this study were as follows: (1) to explore the effect of *P. parvifolia* on the structure and function of the microbial community along an altitudinal gradient in the QLMs, (2) to explore the response of co‐occurrence networks to environmental factors and *P. parvifolia*, and (3) to predict change trends in the effects of *P. parvifolia* on soil denitrification communities during its migration to high altitudes. Our findings on the impact and driving factors of *P. parvifolia* on soil microbial community characteristics and ecological functions will facilitate more effective ecological management strategies in the QLMs in the future.

## 2 MATERIALS AND METHODS

### 2.1 Research area

The study area was located in the Binggou Basin of Qilian County, Qinghai Province (100°11′–100°23′E; 38°02′–38°10′N), which is one of the core areas for ecological protection in China's economic development strategy. The QLMs are located at the intersection of the Qinghai–Tibet Plateau, Mongolian Plateau, and Loess Plateau. It is a sensitive area at the intersection of the monsoon and west winds and has typical continental and plateau climate characteristics. The elevation of the mountainous area fluctuates greatly, the difference in water and heat conditions is considerable, and the vegetation has an evident zonal distribution pattern along the altitudinal gradient (Grytnes et al., 2014; Michael & Warren, 2009). The altitude of the QLMs ranges from 2600 to 4401 m. The average annual temperature is −0.1°C, and the average annual precipitation is 412.6 mm (Zang et al., 2022). The study area is characterized by a continental alpine and subhumid climate, and the typical soil types in the study area are alpine meadow and subalpine shrub meadow soils. *Potentilla parvifolia* is the main shrub in the middle and high altitudes of the QLMs.

### 2.2 Experimental design and soil collection

Soil samples were collected from three elevations (3204, 3550, and 3750 m) in the central QLMs in August 2021. For each elevation, the root zone soil of *P. parvifolia* was used as the experimental object, and bare soil was used as a control. Six sampling sites were selected for this study. Collect soil samples at a depth of 0–10 cm using the plum blossom sampling method, remove the aboveground plants and litter layer (3 cm), mix well, and place them in a sterile aluminum box as a single sample (Ma et al., 2022). The root zone soil was collected by digging out the root system and gently shaking the plant roots, shaking off the attached soil, and wearing sterile gloves to collect the fallen soil blocks (soil 1 cm away from the root system). Follow this step to collect three duplicate samples, and 18 soil samples were collected. For each sample, a portion was air dried, ground, and screened to analyze the physical and chemical properties of the soil (pH, total carbon and nitrogen, ammonium nitrogen and nitrate nitrogen, available phosphorus); partial fresh soil was stored at 4°C for analysis of enzyme activity or −80°C for DNA extraction.

### 2.3 Soil physicochemical analysis

The soil pH was measured using a PHS‐3E pH meter (INESA Scientific Instrument, Shanghai, China) with a 1:5 (m/v) soil‐to‐water ratio. Soil total carbon (TC) and total nitrogen (TN) were determined using an elemental analyzer (Model CN; Vario Macro Elementar, Munich, Germany) (Wang et al., 2017). Extracts were analyzed for ${\mathrm{NH}}_{4}^{+}$–N and ${\mathrm{NO}}_{3}^{-}$–N concentrations using a flow autoanalyzer (Smartchem, Munich, Germany) after extraction with 2 M KCl, following the QuikChem Method (Sennett et al., 2021). Soil available phosphorus (AP) was measured using a spectrophotometer (Mapada Corporation, China) with 0.5 mol/L NaHCO_3_ extraction (Page, 1983).

### 2.4 Soil enzymatic assay

Soil acid phosphatase (ACP) activity was determined using disodium benzene phosphate colorimetry (Tabatabai & Bremner, 1969). Soil urease (URE) activity was determined using the phenol–sodium hypochlorite colorimetric method (Bo et al., 2015). Soil sucrase (SUC) activity was measured using the colorimetric method with 3, 5‐dinitrosalicylic acid (Cai et al., 2019). Soil catalase (CAT) activity was determined through potassium permanganate titration (Trasar‐Cepeda et al., 1999).

### 2.5 DNA extraction and high‐throughput sequencing

Total soil genomic DNA from each soil sample was extracted from 0.5 g soil using a Fast DNA® spin kit (MP Biomedicals, Santa Ana, CA, USA) according to the manufacturer's instructions. The DNA extract was isolated and identified on a 1% agarose gel, and the concentration and purity of the extracted DNA were determined using a NanoDrop UV–Vis spectrophotometer (ND‐2000c, NanoDrop Technologies, Wilmington, DE, USA) (Duan et al., 2021). Bacterial 16S rRNA V3‐V4 hypervariable regions were amplified using the universal primer pair 338F (5′‐ACTCCTACGGGAGGCAGCA‐3′) and 806R (5′‐GGACTACHVGGGTWTCTAAT‐3′), the fungal internal transcribed spacer (ITS)‐targeting primer pair ITS1F (5′‐CTTGGTCATTTAGAGGAAGTAA‐3′), and ITS2R (5′‐GCTGCGTTCTTCATCGATGC‐3′) (Zhang et al., 2020). The PCR volume (20 μL) contained 5× reaction buffer (4 μL), dNTPs (4 μL of 2.5 mM), each primer (0.8 μL of 5 μM), template DNA (1 μL of ca. 10 ng), and 0.4 μL of Fast Pfu DNA Polymerase (TransGen Biotech, Beijing, China), with ultrapure H_2_O to make up to the final volume. 16S rRNA gene amplicon libraries of equal molecular weight were sequenced using the MiSeq platform (Illumina, San Diego, CA, USA) and paired‐end sequencing (2 × 300), according to the standard protocol of Majorbio Bio‐Pharm Technology Co., Ltd. (Shanghai, China). Bacterial and fungal sequences were submitted to the National Center for Biotechnology Information Sequence Read Archive (accession number: SRP426904).

### 2.6 Data analysis

In this study, the FASTQ files were analyzed with the ASV (amplicon sequence variant) method in the ‘DADA1 v10.1.25’ package, using the statistical software R (Callahan et al., 2016). The unique amplified sequence variations (ASVs) were classified using Silva138 (Quast et al., 2013) and UNITE (Nilsson et al., 2019) as reference libraries for bacteria and fungi, respectively. At the quality filtering process, sequence processing was performed in R using the ‘DADA2’ package (Callahan et al., 2016), removing sequences with a quality of <20 and a length of <200 bp (Schloss et al., 2009). Use the mergePairs function of DADA2 to merge forward and reverse sequences, and use the removeBimeraDenovo function of DADA2 to remove chimeric sequence reads. Then combine the ASVs library and taxonomy table for further analysis using the ‘photoseq’ package in R (McMurdie et al., 2013). Before biological statistics, ASVs were rarefied using ‘rarefy_even_depth’ function in package ‘phyloseq’ (McMurdie et al., 2013), and the rarefied ASV table was used to analyze the characteristics of microbial community diversity and composition.

### 2.7 Statistical analysis

The rarefied ASV table was used for microbiological alpha and beta analyses. The alpha diversity of the soil bacteria and fungi was estimated using Chao1 (community richness) and Simpson (community diversity) indices. Principal coordinate analysis (PCoA), which reflects the beta diversity of microbial communities, was performed using QIIME software (v 1.80), and the Bray–Curtis distance matrix was used to assess community similarity (Gregory et al., 2010). A one‐way permutational multivariate analysis of variance (PERMANOVA) was used to determine whether bacterial and fungal communities were significantly influenced by altitude and *Potentilla parvifolia*, with 999 permutations and beta‐group‐significance command in QIIME2 (*p* < .05). Statistical analysis of the alpha diversity indices of bacteria and fungi was performed using Tukey's honestly significant difference test in R (v 3.5.3) (*p* < .05) (Dawen et al., 2019). Analysis of variance (ANOVA) with Duncan's test (*p* < .05) was used to examine the effects of altitude and plants on soil properties and alpha diversity of bacteria and fungi using SPSS 22. Based on the proportional frequencies of DNA sequences from all samples, the relative abundance of the dominant communities of soil bacteria and fungi in each sample was calculated and sequenced at the phylum and order levels. Based on the relative abundance of the bacterial and fungal communities and environmental variables, we calculated the pairwise distances between the samples. Mantel tests were performed to compare the bacterial communities, soil physicochemical properties, and fungal communities. The ecological functions for denitrification of functional bacterial taxa were annotated by the functional annotation of prokaryotic taxa (FAPROTAX) database on the online Majorbio Cloud Platform (https://report.majorbio.com/meta/FAPROTAX/) (Louca et al., 2016). Correlations between the relative abundance of denitrifying functional communities and soil characteristics were determined using redundancy analysis. The soil microbial co‐occurrence network was inferred using sparse correlations for the compositional correlation matrix constructed using the weighted correlation network analysis package in R (v 3.6.1) (Friedman & Alm, 2012; Peter & Steve, 2012). Spearman's correlation analysis was used to calculate the correlation between ASVs, with thresholds of 0.65 and 0.05. In order to ensure sufficient sample size, we combined the ASVs of samples taken at various heights and constructed a network of fungi and bacteria in the bare soil and root zone. Microbial networks were visualized using Gephi software. A set of parameters, including the number of nodes and edges, positive correlation ratio, negative correlation ratio, average degree, and clustering coefficient, were used in this study to describe network topological characteristics. Each node on the networks made up of all the strong correlations represents a different genus, and each edge linking these nodes represents a robust and substantial association (Kyle et al., 2018). The higher the values of network average degree (the average connections of each node with another particular node in the network) and clustering coefficient (the degree to which the nodes tend to cluster together), the closer the network connection (Barberán & Bates, 2012). In addition, by comparing the positive and negative correlation ratios, we assess community stability. It must be noted that stable communities typically have stronger negative cohesion (Hernandez et al., 2021; Yuan et al., 2021). Origin 2021 software was used to draw figures, and the data were presented as the mean ± SD.

## 3 RESULTS

### 3.1 Soil physicochemical properties

The physical and chemical characteristics of the soil at each sampling point are listed in Table 1. *Potentilla parvifolia* increased the TC, TN, and C/N contents in the root zone soil compared with those in the bare soil. At the same time, the TC and C/N were significantly affected by the elevational gradient (*p* < .05), with a tendency to decrease with altitude (Table S1). There was a significant negative correlation between soil pH and altitude, whereas there was no significant difference between the different soils (*p* > .05). There was no significant effect on the AP of *P. parvifolia* (*p* > .05), and the change in AP with altitude was similar to that of pH. At different altitudes, the ${\mathrm{NH}}_{4}^{+}$–N content in the root zone soil decreased significantly (*p* < .05). However, the ${\mathrm{NO}}_{3}^{-}$–N content at 3204 and 3750 m was significantly increased by *P. parvifolia*. ${\mathrm{NH}}_{4}^{+}$–N and ${\mathrm{NO}}_{3}^{-}$–N were simultaneously influenced by *P. parvifolia* and their interactions with environmental factors (Table S1). The average annual temperature in the central part of the QLMs gradually decreased with increasing altitude, whereas the average annual precipitation showed the opposite trend (Table S2).

**TABLE 1:** Soil physicochemical properties among six plots.

Physicochemical properties	LP	LCK	MP	MCK	HP	HCK
pH	6.25 ± 0.11a	6.35 ± 0.08a	6.15 ± 0.06b	6.12 ± 0.04bc	6.02 ± 0.07 cd	6.12 ± 0.04bc
TC (g/kg)	11.56 ± 2.46a	9.43 ± 0.67ab	10.05 ± 3.04ab	6.71 ± 0.54bc	7.05 ± 1.01b	6.35 ± 0.96bc
TN (g/kg)	0.83 ± 0.16a	0.73 ± 0.10a	0.77 ± 0.20ab	0.55 ± 0.04ab	0.64 ± 0.07b	0.56 ± 0.10b
AP (mg/kg)	14.33 ± 2.10a	12.98 ± 3.69a	8.15 ± 0.02bc	9.36 ± 2.07b	4.81 ± 0.25 cd	4.45 ± 0.33d
C/N	13.96 ± 0.38a	12.94 ± 0.99ab	12.92 ± 0.78ab	12.19 ± 0.20b	11.05 ± 0.37c	10.91 ± 0.05c
${\mathrm{NH}}_{4}^{+}$–N (mg/kg)	2.30 ± 0.05d	3.65 ± 0.29a	2.51 ± 0.05 cd	3.10 ± 0.17b	2.72 ± 0.17c	3.43 ± 0.19a
${\mathrm{NO}}_{3}^{-}$–N (mg/kg)	1.77 ± 0.08d	1.47 ± 0.10e	1.91 ± 0.19 cd	1.99 ± 0.27bc	2.23 ± 0.43a	2.08 ± 0.19b

*Note*: Data are expressed as mean ± SD. Significant differences (*p* < .05) among the six sample points were determined using one‐way analysis of variance followed by an LSD test. The same letters indicate no significant differences (a, b, c, d, and e). LP, *P. parvifolia* root zone soil at 3204 m elevation; LCK, bare soil at 3204 m elevation; MP, *P. parvifolia* root zone soil at 3550 m elevation; MCK, bare soil at 3550 m elevation; HP, root zone soil of *P. parvifolia* at 3750 m elevation; HCK, bare soil at 3750 m elevation.

Abbreviations: AP, available phosphorus; C/N, ratio of TC to TN; ${\mathrm{NH}}_{4}^{+}$–N, ammonium nitrogen; ${\mathrm{NO}}_{3}^{-}$–N, nitrate nitrogen; TC, total carbon; TN, total nitrogen.

### 3.2 Soil enzyme activities

The soil enzyme activities in each group are listed in Table 2. Compared with bare land, the enhancing effect of *P. parvifolia* on ACP activity increased with altitude and differed significantly at 3750 m (*p* < .05), whereas the enhancing effect on SUC activity was mainly observed at 3204 and 3750 m. Two‐factor analysis of variance showed that all four enzyme activities were significantly influenced by the altitudinal gradient (Table S1, *p* < .05), and both ACP and CAT activities first increased and then decreased with altitude. Under the same treatment, URE activity increased significantly along the altitudinal gradient. SUC activity was affected by the combination of *P. parvifolia* and environmental factors.

**TABLE 2:** Activities of soil enzymes among six plots.

Enzyme	LP	LCK	MP	MCK	HP	HCK
ACP (nmol/g/h)	172.76 ± 39.08ab	213.94 ± 16.45a	243.40 ± 16.03a	226.02 ± 10.82a	219.60 ± 86.62a	134.70 ± 23.83b
URE (nmol/g/h)	1.014 ± 0.133bc	0.671 ± 0.133c	1.306 ± 0.143b	1.330 ± 0.108b	1.400 ± 0.123b	2.056 ± 0.168a
SUC (nmol/g/h)	710.36 ± 30.50b	209.65 ± 13.92e	487.54 ± 18.26d	508.51 ± 68.22d	990.89 ± 11.33a	597.43 ± 4.44c
CAT (nmol/g/h)	0.363 ± 0.018c	0.315 ± 0.018c	0.624 ± 0.021a	0.621 ± 0.034a	0.466 ± 0.019b	0.519 ± 0.012b

*Note*: Data are expressed as mean ± SD. Significant differences (*p* < .05) among the six sample points were determined using one‐way analysis of variance followed by an LSD test. The same letters indicate no significant differences (a, b, c, d, and e).

Abbreviations: ACP, acid phosphatase; CAT, catalase; SUC, sucrase; URE, urease.

### 3.3 ASV abundance

**FIGURE 1:**
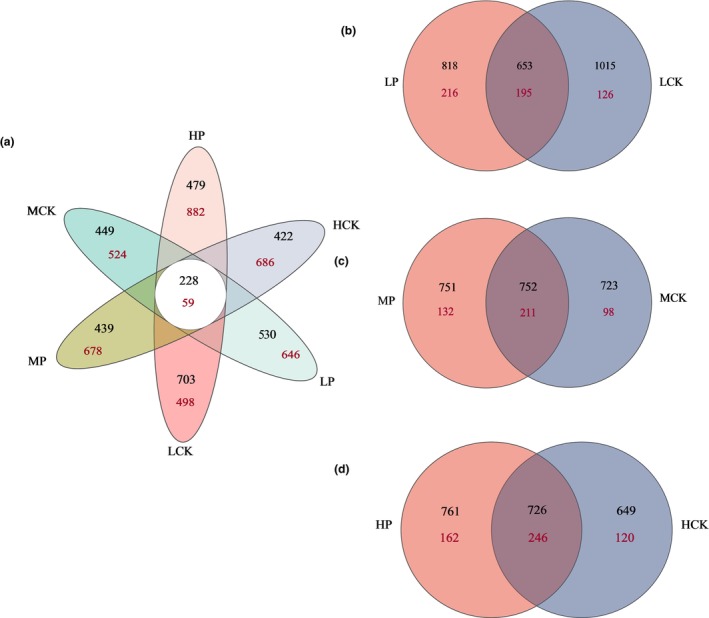
Venn diagram of shared bacterial (black) and fungal (red) amplicon sequence variant across six groups. (a) represents the distribution under the six groups, while (b–d) represent the elevation of 3204, 3550, and 3750 m, respectively.

After removing low‐quality sequences, 1,498,358 bacterial 16S rRNA sequences and 2,108,039 fungal ITS sequences were obtained. Across all samples, 3250 bacterial ASVs and 3973 fungal ASVs were obtained (Figure 1). Compared with fungi, the ASV sequence shared by bacteria was higher in both bare and root zone soils (Figure 1a). Among the fungal ASVs, 59 (1.4%) were common to both soils, whereas 2206 (55.6%) and 1708 (43%) were unique to the root zone soil of *P. parvifolia* and the bare soil, respectively. The ASV sequences common to the bacteria were not significantly different among the three elevations (*p* > .05). At the three elevations, 36, 48, and 47% of the fungal ASVs were common (3204, 3550, and 3750 m, respectively) (Figure 1b–d).

### 3.4 Microbial diversity

#### 3.4.1 Alpha diversity

**FIGURE 2:**
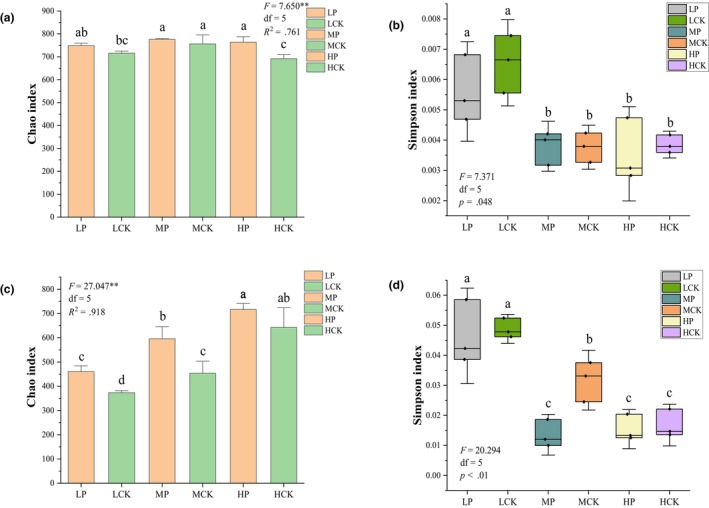
Chao1 and Simpson indices of bacterial (a, b) and fungal (c, d) communities. Data represent means ± SE. The same letters indicate no significant differences (a–c).

The alpha diversity indices of the soil bacterial and fungal communities in the six plots are shown in Figure 2. The Chao1 index of soil bacteria was affected by the combination of *P. parvifolia* and altitudinal gradient (Table 3). *Potentilla parvifolia* increased the community richness of root zone bacteria, especially at 3750 m (Figure 2a, *p* < .05, *R* ^2^ = .761). The Simpson index of bacteria was mainly controlled by altitude and gradually decreased along the altitudinal gradient (Figure 2b). The richness and diversity indices of fungi were positively correlated with altitude, which was similar to the results for bacteria, whereas the positive effect of *P. parvifolia* on the alpha diversity of fungi was mainly observed at 3204 and 3550 m, and the difference was not significant at 3750 m (Figure 2c,d, *p* > .05). Spearman correlation analysis showed that bacterial and fungal diversity was positively correlated with precipitation and enzyme activity but negatively correlated with soil nutrient content and temperature (Figure S1).

**TABLE 3:** Double factor variance analysis of microbial alpha diversity.

Parameters	Bacterial community		Fungal community	
	Chao1	Simpson	Chao1	Simpson
	*p*_value	*p*_value	*p*_value	*p*_value
Altitude	<.05	<.01	<.01	<.01
*Potentilla parvifolia*	<.01	n.s	<.01	<.05
Altitude and *P. parvifolia*	n.s	n.s	n.s	n.s

Abbreviation: n.s, no significant difference.

#### 3.4.2 Beta diversity

**FIGURE 3:**
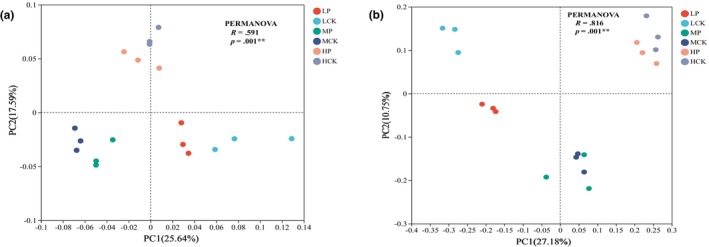
Principal coordinate analysis based on the Bray–Curtis distance matrix for six groups. (a, b) represent the bacterial and fungal communities, respectively. Points with different colors or shapes represent samples from different groups. The closer the two sample points are, the more similar their species composition of the two samples.

The results of the microbial beta diversity analysis are shown in Figure 3. PERMANOVA revealed significant differences at the level of bacterial and fungal microbial communities (*p* < .01). The PCoA of bacteria showed that the sample points of the six groups were clearly separated, indicating that the differences in the bacterial communities were caused by *P*. *parvifolia* and altitude (Figure 3a, *r* = .591). The PCoA of fungi indicated that both soil microbial communities were clearly separated at 3204 m, whereas they clustered more closely at 3550 and 3750 m. Fungal communities differed significantly among the elevations (Figure 3b, *r* = .861).

### 3.5 Microbial community composition

**FIGURE 4:**
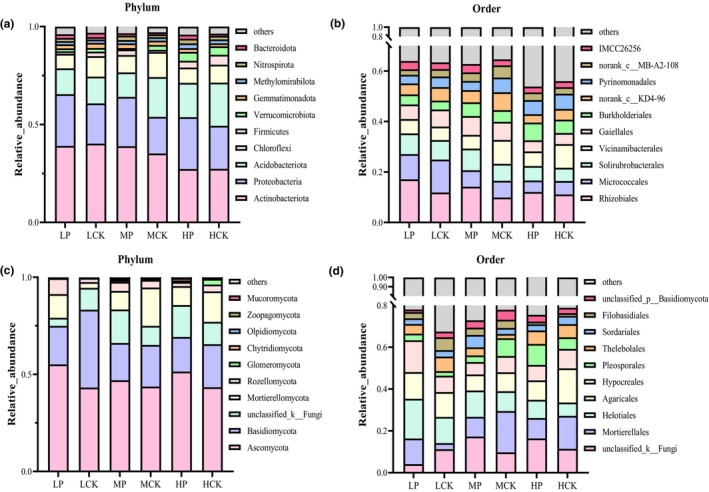
Relative abundance (%) of bacterial and fungal communities of six groups. (a, b) represent phylum and order level of bacteria, respectively. (c, d) represent phylum and order level of fungi, respectively.

We assessed the taxonomic distribution of the bacterial and fungal ASVs at different classification levels (Figure 4). For bacteria, *Actinobacteriota*, *Proteobacteria*, and *Acidobacteriota* were the dominant phyla in all the groups (Figure 4a). The relative abundance of *Actinobacteria* decreased gradually with altitude, whereas the effect of *P. parvifolia* was not significant (*p* > .05). Correlation analysis showed that the relative abundance of *Actinobacteriota* positively correlated with pH and nutrient content (Figure S2). *Potentilla parvifolia* increased the relative abundance of *Proteobacteria*. At the order level, the relative abundance of Rhizobiales was significantly higher in the LP (22%) and MP (19%) groups than in the LCK (16%) and MCK (13%) groups (Figure 4b, ANOVA, *p* < .05). The relative abundance of Micrococcales was highest at 3204 m and gradually decreased with altitude. Among fungi, *Ascomycota*, *Basidiomycota*, and *Mortierellomycota* were the dominant phyla (Figure 4c). The relative abundance of *Ascomycota* in the root zone soil of *P. parvifolia* was significantly higher than in the bare soil at the same altitude. *Basidiomycota* had the highest relative abundance in the LCK group (30%), but the differences were not significant among the other groups (*p* > .05). *Ascomycota* was negatively correlated with pH and ${\mathrm{NH}}_{4}^{+}$–N, whereas *Basidiomycota* showed the opposite trend (Figure S2). At the order level, the relative abundance of Helotiales significantly decreased with increasing altitude. Mortierellales was more abundant in the MCK and HCK groups than in the other groups (Figure 4d).

### 3.6 Correlation between microbial community structure and environmental factors

**FIGURE 5:**
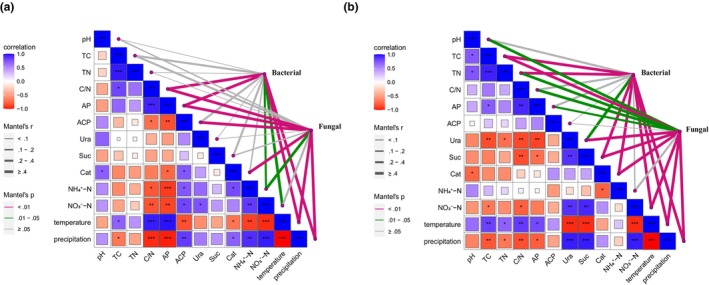
Environmental factor pairwise comparisons are displayed with a color gradient heatmap indicating Pearson's correlation coefficients. The composition of bacterial and fungal community was related to (a) root zone soil properties and (b) bare soil properties using Mantel tests. Edge color indicates statistical significance, and edge width reflects the Mantel's *r* statistic for the corresponding distance correlations.

To analyze the environmental drivers of soil microbial community structure and the mechanism of the effect of *P. parvifolia*, we connected distance‐corrected differences in root zone and non‐root zone soil microbial compositions with environmental factors. The results showed that C/N, nitrate nitrogen, temperature, and precipitation were significantly correlated with the bacterial and fungal community structure under both soils. Compared with that in bare land, the correlation of the microbial community structure in the root zone of *P. parvifolia* with TC, TN, soil enzyme activity, and pH was weakened, whereas the correlation with AP and ${\mathrm{NH}}_{4}^{+}$–N was enhanced (Figure 5). In addition, bacterial and fungal communities are significantly correlated with climate factors, while fungal communities are also significantly affected by soil available phosphorus and soil enzyme activity.

### 3.7 Bacteria FAPROTAX analysis

**FIGURE 6:**
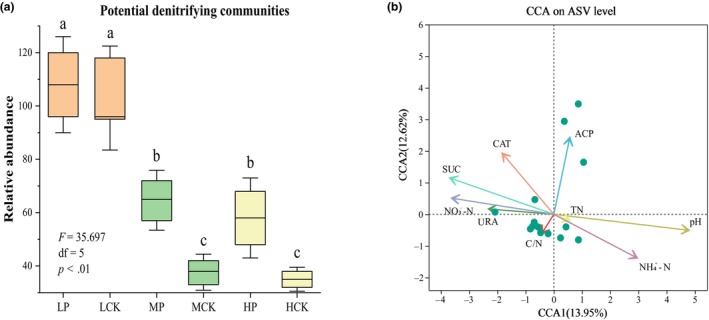
FAPROTAX function prediction of 16S amplicon sequencing results was performed. The relative abundance of potential denitrification communities in different groups (a) and redundancy analysis of the relationship among soil physicochemical properties, denitrification communities, and enzyme activities (b). The same letters indicate no significant differences (a–c).

This study found that the community composition and ecological function of soil microorganisms were affected by a combination of *P. parvifolia* and environmental factors. FAPROTAX analysis revealed that the relative abundance of denitrification community decreased with altitude, and there was significant difference between 3204 and 3550 m (Figure 6a, *f* = 35.697, *p* < .01). Compared to bare soil, the relative abundance of denitrification community was increased by *P. parvifolia*, and the difference was significant at 3550 and 3750 m. The results of the redundancy analysis showed that ACP, SUC, ${\mathrm{NH}}_{4}^{+}$–N, ${\mathrm{NO}}_{3}^{-}$–N, and soil pH were the main explanatory variables affecting the potential denitrification community (Figure 6b). Among these factors, soil pH had the greatest effect.

### 3.8 Microbial co‐occurrence network

**FIGURE 7:**
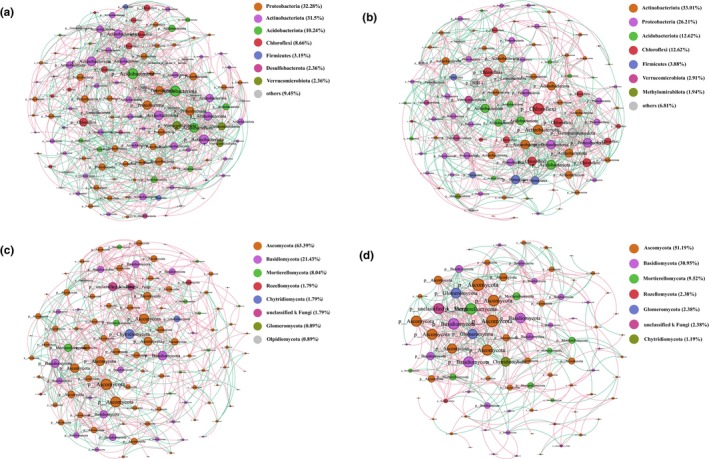
Co‐occurrence network of bacterial amplicon sequence variant (ASVs) (a, b) and fungal ASVs (c, d). (a, c) represented the cover soil of *Potentilla parvifolia*, while (b, d) represented the bare soil. The size of each node is proportional to the connectivity of the ASV; nodes with the same color belong to the same phylum; the links in red color represent positive interaction, and those in green represent negative interaction. The thickness of each edge is proportional to the magnitude of the correlation coefficient (Spearman *r* > ±0.6 and *p* < .05).

By employing co‐occurrence network analyses of the bacterial and fungal sequence data, we built a microbial network of QLMs and investigated their topological properties (Figure 7). The bacterial network consisted of 230 nodes connected by 1536 edges. However, the fungal network had fewer edges (980) and nodes (196) than the bacterial network. The bacterial network had a higher percentage of negative links than the fungal network in both soils (Table S3). *Actinobacteriota*, *Proteobacteria*, and *Acidobacteriota* were the dominant soil bacteria, accounting for 74% (Figure 7a) and 71.8% (Figure 7b) of the total species abundance, respectively. *Acidobacteriota* and *Proteobacteria* were the key groups in the microbiological network of the root zone soil, whereas *Actinobacteria* and *Chloroflexi* were the key groups in bare soil. Compared with the control, the average degree, number of nodes, and edges of the root zone soil network were larger, indicating that the network complexity was higher. In addition, an unclassified phylum, *NB1‐j*, was a key group in the network, although its abundance was only approximately 1%. For fungal network, the average degree, cluster coefficient, and negative correlation ratio of the root zone soil were much higher than those of bare soil, indicating that the interaction and coexistence of species were promoted by *P. parvifolia* (Figure 7c,d, Table S3). *Ascomycota*, *Basidiomycota*, and *Mortierellomycota* were not only the dominant phyla in both soils but also the key groups in the network. *Chytridiomycota* was the key network nodes in the root zone soil (Figure 7c), while *Glomeromycota* was the key nodes in the microbial network of bare soil (Figure 7d).

## 4 DISCUSSION

### 4.1 Soil physicochemical properties and enzyme activities in different habitats

In alpine meadow areas, the soil properties at different altitudes are regulated by the interaction between vegetation cover and climate factors, mainly through the regulation of soil biogeochemical cycles (Deyn & Putten, 2005). The response of these basic nutrient cycles to the environment leads to differences in nutrient availability between elevations and, in turn, affects the resource availability and habitat conditions of the soil microbial community (Dinakaran et al., 2018; Guerrero‐Ramirez et al., 2020). The results showed that *P. parvifolia* had a positive effect on the soil nutrient content and availability (Table 1). The TC and TN concentrations in the root zone soil were significantly higher than those in the bare soil. As the dominant shrub in the QLMs, *P. parvifolia* has important effects on soil properties and microenvironment (Drewnik et al., 2016; Tai et al., 2013). In general, larger shrub types have strong nutrient accumulation capacities (Klein et al., 2007; Science – Soil Science and Hydrology, 2019), which may contribute to the growth and enrichment of root zone‐dominant microbial community (Gao et al., 2021; Ye et al., 2014). In addition, *P. parvifolia* increased the soil C/N ratio, which is conducive to nutrient fixation by root zone soil microorganisms. This study found that the soil nutrient content and pH were negatively correlated with altitude (Table S1, *p* < .01), which is consistent with the findings of Yang, Li, et al. (2022). The correlation analysis results showed that nutrient content was significantly negatively correlated with precipitation and positively correlated with temperature (Figure 5). Low temperatures at high altitudes affect water viscosity and membrane permeability, often inhibiting microbial activity and ultimately reducing C and N content and nutrient availability (Reich & Oleksyn, 2004). Available P content showed the same trend as soil pH. As pH increases, soil P adsorption weakens, thereby increasing P availability (Bair & Davenport, 2012). In contrast, we found a significant positive correlation between soil ${\mathrm{NO}}_{3}^{-}$–N content and altitude, whereas ${\mathrm{NH}}_{4}^{+}$–N was mainly influenced by *P. parvifolia* (Table S1). This differs slightly from the findings of Melillo et al. (Melillo et al., 2011) in temperate forests. The abundant vegetation at low altitudes possibly promotes denitrifying microbial activity and increases gaseous N loss, thereby reducing soil N availability (Tang et al., 2018). The low ${\mathrm{NH}}_{4}^{+}$–N content in the root zone soils may be related to the promotion of nitrification by plants.

Soil enzymes are important drivers of soil nutrient metabolism and affect the soil microbial numbers and community structure (Lehmann et al., 2011; Trasar‐Cepeda et al., 2000). *Potentilla parvifolia* was significantly correlated with soil ACP and SUC activities, where altitude was significantly correlated with the activities of the four enzymes (Table S1). *Potentilla parvifolia* increased URE and SUC activities in the root zone soil at 3204 m (Table 2). With increasing altitude, enzyme activity was co‐regulated with climate factors, and plants showed different trends. URE activity increased with elevational gradient, whereas CAT and ACP activities first increased and then decreased. With increasing altitude, high precipitation provides abundant water for plants and promotes plant growth and soil carbon input, leading to an increase in soil enzyme activity (Yang, Feng, et al., 2022). In addition, lower soil temperatures reduce soil enzyme activity to a certain extent (Jin et al., 2009). Thus, temperature and precipitation jointly affect soil enzyme activity (Edwards et al., 2006).

### 4.2 Driving factors of soil bacterial community characteristics

As an important part of the soil ecosystem, bacteria promote material exchange between plant roots and soil (Zhang et al., 2016). The research results showed that there were differences in the effects of *P. parvifolia* on soil bacterial community characteristics at different altitudes. *Potentilla parvifolia* significantly increased the soil bacterial richness (Chao1) index, particularly in the 3750 m altitude (Figure 2a). The increasing of bacterial community richness in the root zone soil was related to the increase in soil TC and TN content mediated by plants. At high altitudes, suitable climate conditions promote the decomposition and release of the rhizosphere litter of *P. parvifolia* and lead to changes in bacterial richness by affecting bacterial metabolism and soil enzyme activity (Bais et al., 2006). The altitude gradient was significantly correlated with bacterial diversity (*p* < .01), and the *P. parvifolia* had no significant effect on it (Table 3). The results showed that the diversity of the soil bacteria gradually increased with altitude (Figure 2b) and was significantly positively correlated with precipitation (Figure S1), which is supported by the results of Maryam et al. (2022). Humid climates are conducive to the coexistence of diverse species. In addition, there were no significant differences in the bacterial diversity between root zone of *P. parvifolia* and the control at any altitude, indicating that *P. parvifolia* mainly affected the bacterial richness index. PCoA showed that *P. parvifolia* caused a significant difference in the bacterial community (Figure 3a), which was related to the improvement in soil properties and enzyme activities by plants. The significant differences in the similarity distances between the soil bacterial communities indicate that transmission constraints play an important role in the altitudinal patterns of bacterial communities. This limiting effect may be due to more unstable environmental conditions along the altitudinal gradient, leading to the competitive exclusion of bacterial communities (Woodcock et al., 2006).

The overall composition of microbial communities may differ greatly along the elevational gradient in the QLMs in China, whereas their dominant groups remained mostly similar in terms of relative abundance. This finding was similar to that of Yuan et al. (2017). *Actinobacteriota*, *Proteobacteria*, *Acidobacteriota*, and *Chloroflexi* dominated the soil bacterial communities in the different habitats (Figure 4a). The results showed that *P. parvifolia* favored the growth and enrichment of *Proteobacteria*, whose relative abundance was strongly correlated with precipitation (Figure S2). Abundant water resources make root zone soil‐dominant bacteria more concentrated (Zhang, Vivanco, & Shen, 2017). The relative abundance of *Actinobacteriota* was regulated by both plant species and altitude. As one of the beneficial bacteria in soil, *Actinomycetes* mainly decompose organic matter, and the decreasing trend of their abundance with altitude was related to soil nutrients. However, *P. parvifolia* mitigated this trend. Soil bacterial community distribution was closely related to environmental factors such as soil elemental content, temperature, and precipitation (Figure 5), which conforms to the environmental selection view of the spatial distribution of microbial communities (Chen et al., 2017; Dharmesh et al., 2012). However, along the altitudinal gradient, the bacterial community composition was significantly correlated with the C/N ratio, indicating that substrate restriction regulated changes in the soil bacterial community structure (Sarto et al., 2020).

In complex ecosystems, ecological network analysis is used to study the interaction mechanisms of microorganisms and stability of microbial systems based on the random matrix principle, which can be used to predict the functions of soil ecosystems (Fuhrman, 2009; Ye et al., 2012). We found that the number of nodes and edges and average connectivity of the root zone soil microbial network were higher than those of the control (Figure 7a,b, Table S3), whereas the proportion of positive correlations was not significantly different from that of bare soil. The root zone microbial network is more complex, which may be related to increased bacterial community richness and synergistic interactions among soil bacteria.

### 4.3 Driving factors of soil fungal community characteristics

The effect of *P. parvifolia* on fungal community characteristics also varied along the elevational gradient. Compared with the control, *P. parvifolia* significantly increased the richness and diversity indices of soil fungi, especially at 3204 and 3550 m (Figure 2c,d). The results of the correlation analysis showed that the fungal community structure was significantly correlated with soil AP contents (Figure 5). Some studies have reported a strong correlation between the richness of soil fungal communities and specific plant species (Urbanová et al., 2015). Many soil fungi are specific root symbionts and pathogens, and their survival is directly dependent on the interaction between root litter and tree biotrophy (Wardle, 2006). In addition, the diversity of soil fungi is simultaneously regulated by factors related to altitude (Table 3), and their distinct vertical distribution patterns result from spatial heterogeneity in soil properties and climate conditions (Marinari et al., 2005). The present study found that fungal alpha diversity had a relatively distinct pattern of elevation and was concluded to have a strong negative relationship with soil C/N (Figure S1), consistent with the findings of Ogwu et al. (2019) and Yingying et al. (2018). The soil C/N ratio is generally considered to represent nutrient availability (Cleveland & Liptzin, 2007). Thus, the soil C/N ratios in different habitats can significantly influence fungal anabolism and foraging strategies (Grosso et al., 2016). Several studies have found that a high C/N ratio may disrupt the stoichiometric balance between the soil and hyphae, inhibiting the activity of exocrine enzymes and the accumulation of fungal biomass (Sinsabaugh et al., 2013). The results of the PCoA analysis showed that the effect of *P. parvifolia* on the similarity of fungal communities was mainly concentrated at 3204 m. Yuichiro et al. (2010) found that the net primary productivity of *P. parvifolia* is positively correlated with temperature and could act as a major contributor to CO_2_ deposition. Temperature continuously decreased along the elevational gradient, resulting in a weakening of the effect of *P. parvifolia* on the fungal community, accompanied by a decrease in productivity. In the altitudinal gradient pattern, the fungal community similarity showed a distance‐attenuated biogeographical distribution (Figure 3b).

In the present study, *Ascomycota*, *Basidiomycota*, and *Mortierellomycota* were the dominant phyla at all sampling sites (Figure 4c). *Potentilla parvifolia* significantly promoted the growth and enrichment of *Ascomycota*. *Ascomycetes* are not only the main decomposers of organic matter in soil fungal communities but also form symbiotic mycorrhizas with plants, participating in the root zone nitrogen cycle (Anna et al., 2009). In addition, *Basidiomycota* and *Mortierellomycota* were positively and negatively correlated with pH, respectively. The relative abundance of *Mortierellomycota* increased with elevation, which may be related to its strong adaptation to the harsh soil environments at higher altitudes (Li et al., 2018).

Compared with that of bacteria, the fungal microbial network had a fewer number of nodes and edges, average connectivity, and negative correlation ratio, which indicated that the interaction between fungal communities and the network stability was weaker (Figure 7c,d). The ratio of positive to negative associations affects the stability of microbial communities (Herren & McMahon, 2017). *Ascomycetes*, *Basidiomycetes*, and *Mortierellomycota* were key groups in each network. In addition, *Chytridiomycota* and *Olpidiomycota* were the key nodes of the root zone soil microbial network, whereas *Glomeromycota* and *unclassified k‐fungi* were the key nodes of the bare soil microbial network. The results showed that *P. parvifolia* changed the soil microenvironment and affected interactions between fungal communities. From the topological properties of the network, we can see that the complexity and stability of the root zone soil microbial network were much higher than those of bare land. In general, *P. parvifolia* had a greater influence on the fungal symbiotic networks than on the bacterial networks.

### 4.4 Relationships among *P. Parvifolia*, environmental factors, and denitrification

Microbial‐mediated denitrification is an important component of the nitrogen cycle in alpine meadow ecosystems and is directly related to soil nitrogen availability (Liming et al., 2021). Additional carbon sources and environmental factors (pH, ${\mathrm{NO}}_{3}^{-}$–N, and water) determine the niche differentiation of denitrifying microorganisms, thereby affecting the denitrification process (Surey et al., 2020; Xu et al., 2016). In the present study, we performed functional predictions from the 16S sequencing results. The results showed that the relative abundance of potential denitrifying community in root zone soil was increased compared with the control, and the change was significant at 3550 and 3750 m (Figure 6a). *Potentilla parvifolia* promotes nutrient cycling and increases soil nutrient utilization by decomposing litter, which is beneficial for the growth and reproduction of denitrifying functional groups (Hui‐Juan et al., 2014). Environmental factors are also involved in regulation. The relative abundance of denitrifying community decreased with the elevational gradient, which was similar to the trends in soil pH, temperature, and TN. A lower pH in acidic soils can limit the growth of denitrifying bacteria while reducing the availability of organic carbon and mineral nitrogen. ${\mathrm{NH}}_{4}^{+}$–N and ${\mathrm{NO}}_{3}^{-}$–N, as initial substrates in the nitrification and denitrification processes, affect the growth and activity of denitrifying bacteria (Zhanming et al., 2022). The results of redundancy analysis also supported this observation (Figure 6b). It is important to note that denitrifying 16S rRNA amplicon sequencing revealed a relatively low frequency of ASVs in the bacterial group. In comparison with metagenomic sequencing techniques, FAPROTAX analysis, a quick and convenient tool for predicting the function of bacterial taxonomic groups, has gaps in accuracy and coverage.

This study assessed the potential denitrification communities based on FAPROTAX predictions, revealing a significant influence of both altitude gradient and *P. parvifolia* on the denitrification process. However, several limitations should be acknowledged. Firstly, the evaluation of denitrification was solely reliant on predictive models of microbial community function, which may introduce uncertainties. Secondly, the study relied on a single time‐point sampling, which limits the robustness and generalizability of our findings. Moreover, it is important to recognize that as altitude increases, many confounding factors, including variations in soil texture and geographical characteristics, can affect the measured parameters. In future research endeavors, we aim to mitigate these limitations by employing in situ detection technologies in conjunction with controlled indoor experiments, facilitating a more comprehensive exploration of the mechanisms underlying these influencing factors.

## 5 CONCLUSION

*Potentilla parvifolia*, a dominant shrub in the QLMs, has varying effects on microbial communities at different altitudes. At 3204 m, *P. parvifolia* mainly affected the fungal community composition and diversity. The accumulation of soil nutrients in the root zone increased the community richness of fungi and relative abundance of the dominant phylum (*Ascomycota*). At 3550–3750 m, *P. parvifolia* significantly affected bacterial community characteristics by increasing bacterial community richness, and enriching the relative abundance of denitrification community. Climate factors and soil nutrient availability jointly drive the evolution of microbial communities along altitudinal gradients. In addition, compared to bare soil, the root zone microbial network has higher stability and complexity, which is most obvious in the fungal community. In the future, with climate change, *P. parvifolia* will continue to migrate to high‐altitude areas and have a more profound impact on bacterial communities with important ecological functions, especially the denitrifying community.

## AUTHOR CONTRIBUTIONS

**Miaomiao Cheng:** Data curation (equal); writing – original draft (equal). **Jinge Song:** Writing – review and editing (equal). **Weikun Li:** Methodology (equal). **Yiming Zhao:** Methodology (equal). **Gaosen Zhang:** Formal analysis (equal). **Yong Chen:** Writing – review and editing (equal). **Haining Gao:** Funding acquisition (equal).

## FUNDING INFORMATION

This work was supported by the Scientific Technology Research Projects of Gansu Province (22JR5RG564, 20YF3WA024), Hexi University Hexi Biodiversity Conservation and Utilization Research Center, Scientific Technology Research Projects of Lanzhou City (2017‐4‐102), Fundamental Research Funds for the Central Universities (lzujbky‐2021‐kb05), Assurance Project of Ecological Planting and Quality of Daodi Herbs (Science and Technology Department of the State Administration of Traditional Chinese Medicine (2020) No. 153), and Science and Technology Bureau of Chengguan District (2017KJGG0057).

## CONFLICT OF INTEREST STATEMENT

The authors declare that they have no competing interests.

## Supporting information


Appendix S1

## Data Availability

The datasets analyzed during the current study are available in the National Center for Biotechnology Information Sequence Read Archive (accession number: SRP426904).
