# Supplementary material for: Potentilla parvifolia strongly influenced soil microbial community and environmental effect along an altitudinal gradient in central Qilian Mountains in western China
Source: Ecol Evol. 2023 Nov 14;13(11):e10685. doi: 10.1002/ece3.10685 (PMC10645544; doi:10.1002/ece3.10685)
Supplement: Supplementary file 1 — Appendix S1 [file ECE3-13-e10685-s001.docx]

Supplementary information

*Potentilla parvifolia* strongly influenced soil microbial community and environmental effect along an altitudinal gradient in central Qilian Mountains in western China

*Miaomiao Cheng^a,b,1^*, *Jinge Song^c,1^*, *Weikun Li^b^*, *Yiming Zhao^b^*, *Gaosen Zhang^d^, Yong Chen^b,*^, Haining Gao^a,*^*

^a^ Key Laboratory of the Hexi Corridor Resources Utilization of Gansu, Zhangye, Lanzhou 734000, China

^b^ School of Life Sciences, Lanzhou university, Lanzhou 730000, China

^c^ School of Stomatology, Lanzhou university, Lanzhou 730000, China

^d^ Key Laboratory of Extreme Environmental Microbial Resources and Engineering, Lanzhou 730000, China

***Correspondence:**

Y. Chen, School of Life Sciences, Lanzhou University, 730000, China. E-mail addresses: [chenyong@lzu.edu.cn](mailto:chenyong@lzu.edu.cn).

H. Gao, Key Laboratory of the Hexi Corridor Resources Utilization of Gansu, Zhangye, 734000, China. E-mail addresses: [gaohn2004@163.com](mailto:gaohn2004@163.com).

*^1^*These authors contributed equally to this work and share first authorship.

Contents:

1: Table S1: Effects of *Potentilla parvifolia*, altitude, and their interactions on the chemical characteristics in soil.

2: Table S2: Climatic conditions of sampling points at different altitudes.

3: Table S3: Microbial network topology parameters.

4: Fig. S1: Correlation analysis of soil microbial diversity with soil properties and climatic factors.

5: Fig. S2: Correlation analysis between the relative abundance of soil microbial dominant phyla and soil properties and climatic factors.

**Table S1** Effects of *Potentilla parvifolia*, altitude, and their interactions on the chemical characteristics in soil


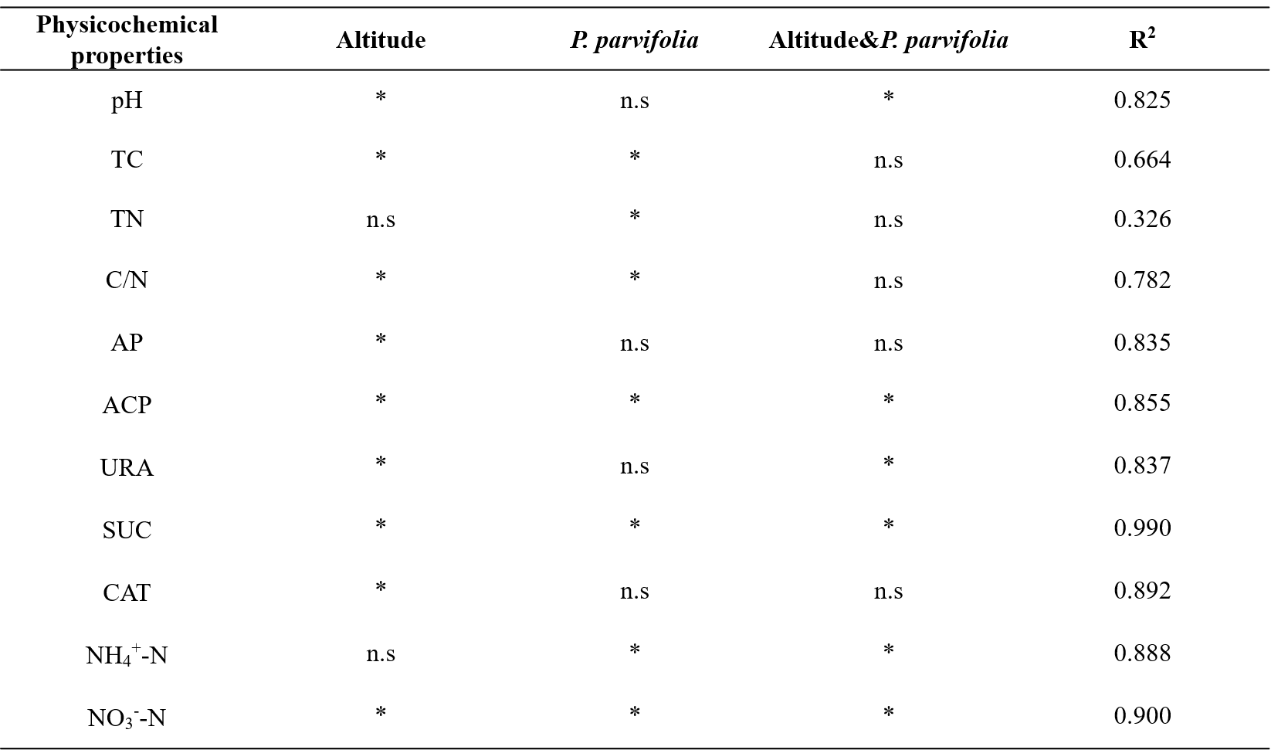


Notes: n.s represents no significant difference. * indicates significance at the 0.05 probability level. ** indicates significance at the 0.01 probability level.

**Table S2** Altitude distribution and Climatic characteristics of the six sampling sites


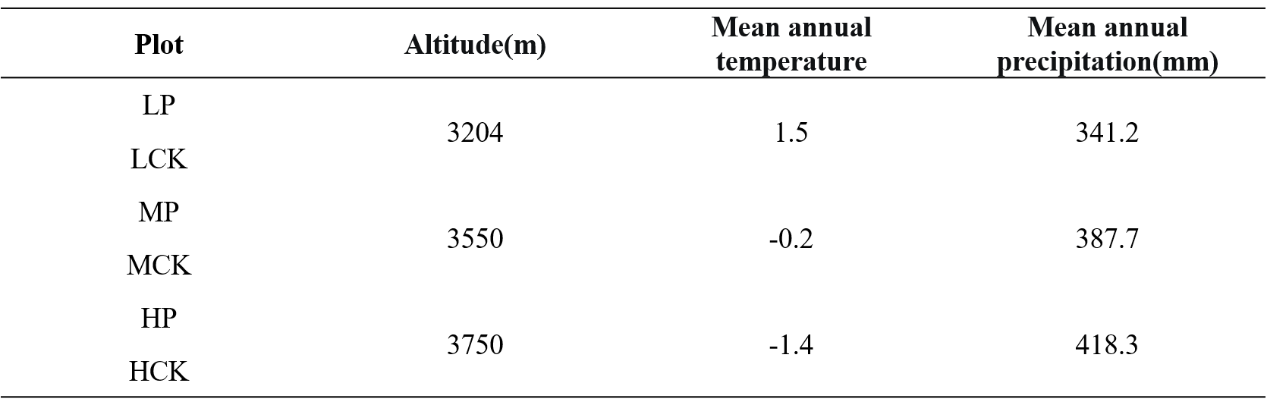


**Table S3** Topological parameters of the collinear network of rhizosphere and non-rhizosphere microorganisms.


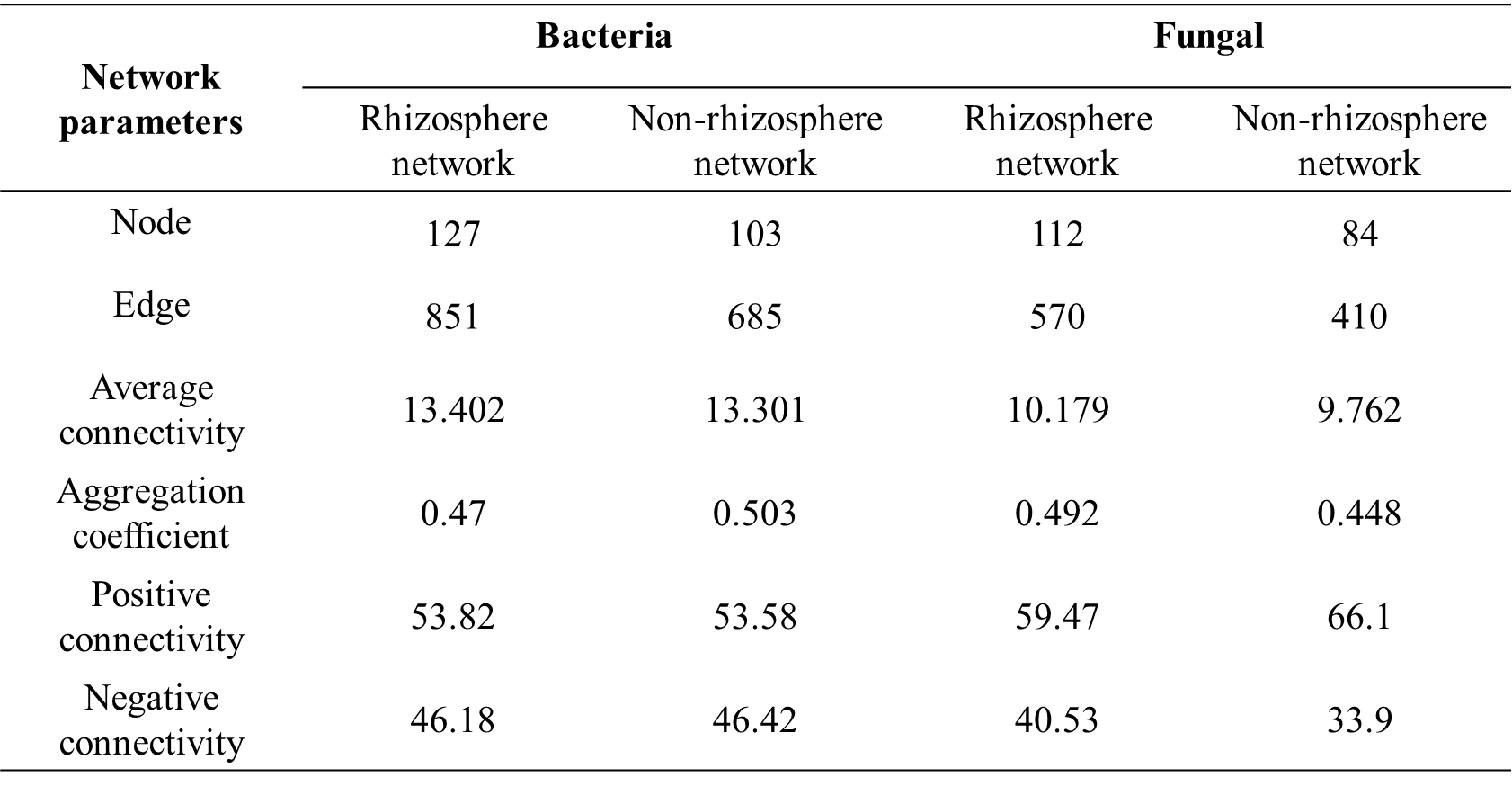


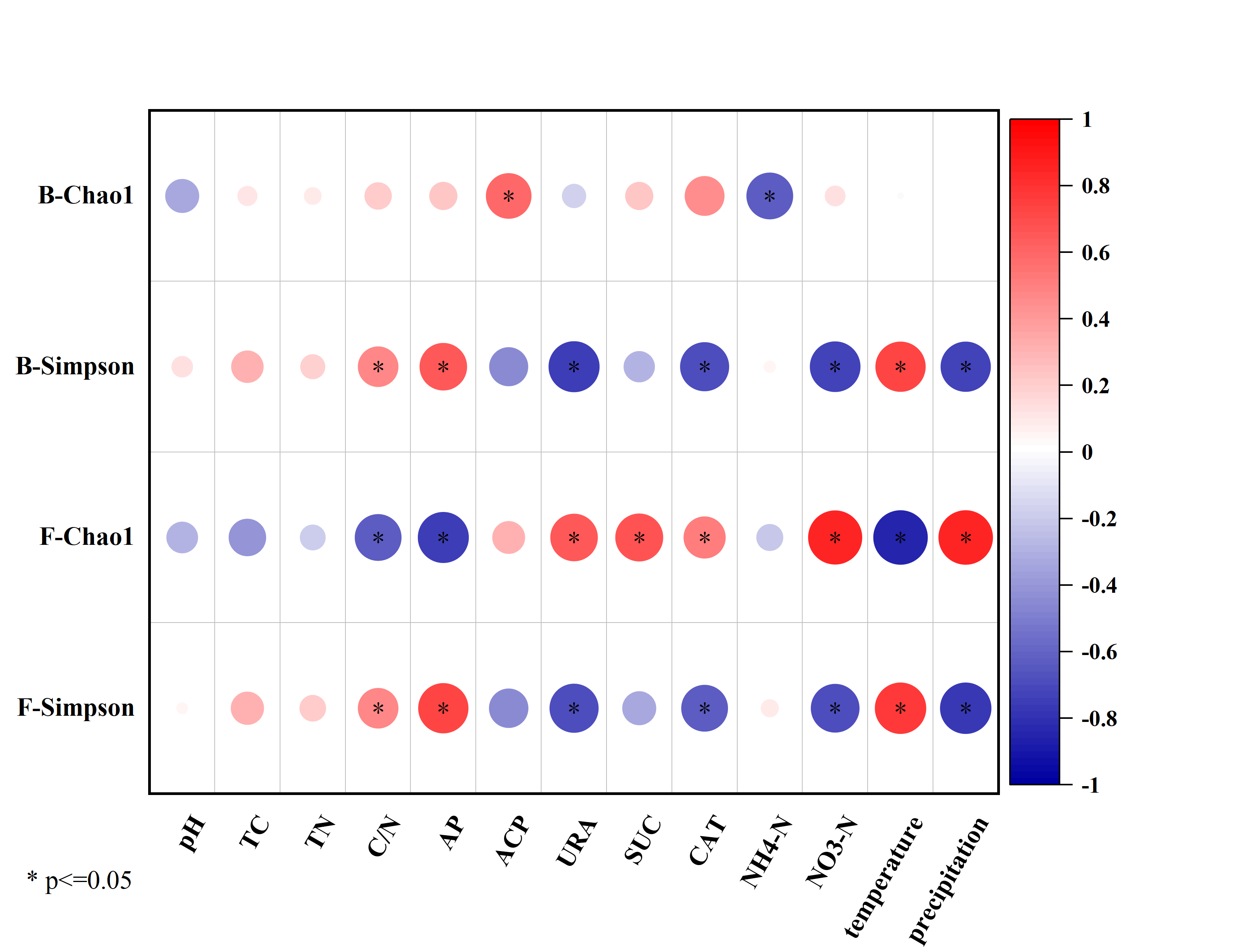


**Fig. S1** Correlation Heatmap of soil physicochemical indexes with bacterial diversity (B-Chao1、B-Simpson) and fungal diversity (F-Chao1、F-Simpson). The size of circle represents the variable’s importance. Spearman correlations are indicated by the color.


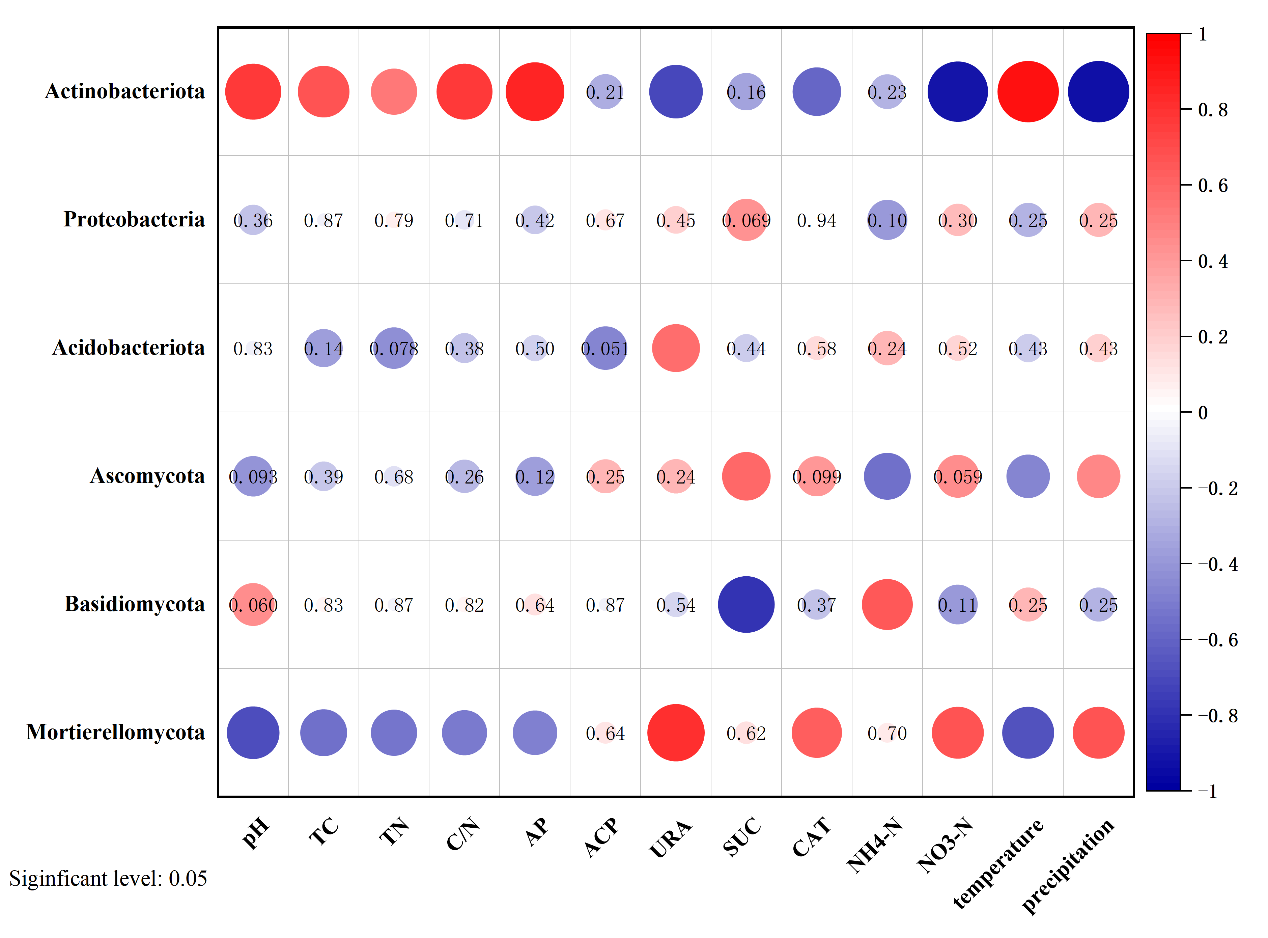


**Fig. S2** Correlation Heatmap of soil physicochemical indexes with the relative abundance of bacterial dominant phyla (*Actinobacteria*, *Proteobacteria*, and *Acidobacteriota*) and fungal dominant phyla (*Ascomycota*, *Basidiomycota*, and *Mortierellomycota*).
